# Meta-analysis of Long-Term Relapse Rate of Type 2 Diabetes Following Initial Remission After Roux-en-Y Gastric Bypass

**DOI:** 10.1007/s11695-021-05692-4

**Published:** 2021-09-10

**Authors:** Zhiqing Yu, Peiwu Li, Peirong Li, Haidan Zhang, Youcheng Zhang

**Affiliations:** 1 Emergency Center, Lanzhou University Second Hospital, Lanzhou, 730030 Gansu China; 2 General Surgery, Lanzhou University Second Hospital, Lanzhou, 730030 Gansu China

**Keywords:** Type 2 diabetes, Bariatric surgery, Roux-en-Y gastric bypass, Sleeve gastrectomy, Meta-analysis

## Abstract

This study aims to determine the long-term relapse rate of type 2 diabetes (T2DM) following initial remission after Roux-en-Y gastric bypass (RYGB) surgery. We searched studies in PubMed, Embase, and the Cochrane Library. A total of 17 eligible studies were included for analysis. Meta-analysis suggested a pooled long-term relapse rate of 0.30 (95% confidence interval [CI], 0.26–0.34) and a remission rate of 0.63 (95% CI, 0.55–0.72) after RYGB and a hazard ratio of 0.73 (95% CI, 0.66–0.81) for comparison of RYGB and sleeve gastrectomy (SG). Subgroup analyses established pooled results. This study suggested RYGB may be a preferred regime for obese patients with T2DM because it is associated with lower long-term relapse and relatively higher initial remission and was also superior to SG due to lower risk of recurrence.

**Graphical Abstract:**

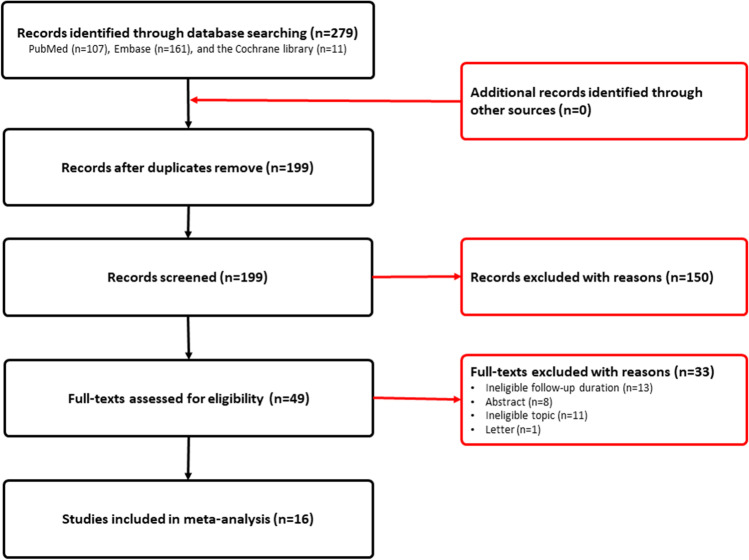

**Supplementary Information:**

The online version contains supplementary material available at 10.1007/s11695-021-05692-4.

## Introduction

Type 2 diabetes mellitus (T2DM) is a chronic and life-threatening disease, which is associated with increased risk of cardiovascular disease and microvascular or macrovascular complications if it will not be well controlled [1]. Issued data suggested that 382 million people were identified with diabetes worldwide in 2013 and this figure is estimated to increase to 592 million by 2035 [2]. Evidence revealed a strong association between obesity and T2DM [3]. Unfortunately, conventional treatments such as medical and excise therapies do not achieve satisfactory outcomes among obese patients with diabetes [4–6]. Specifically speaking, more than half of obese patients with diabetes do not achieve therapeutic goal after receiving conventional treatment regimes [7, 8]. It must be noted that T2DM patients accompanied by obesity will encounter higher medical expenditures, poor quality of life (QoL), and increased mortality after experiencing serious complications and adverse events (AEs) if remission was not obtained [1]. In contrast, remission of T2DM and obesity will reduce the risk of subsequent vascular conditions [9, 10]. Therefore, more aggressive therapies are needed to effectively treat patients with severe obesity and long-standing T2DM [11].

Among several aggressive therapeutic regimens, bariatric surgery has been currently regarded as the preferred option to treat obese patients with T2DM to date [12–14]. Published data reported that, in fact, a half million bariatric surgeries are being performed annually worldwide [15]. Most importantly, Roux-en-Y gastric bypass (RYGB) and sleeve gastrectomy (SG) have become the two most frequent bariatric surgical procedures in recent years [16]. Studies reported that the remission rate was ranging from 38 to 75% after receiving RYGB surgery [17, 18]. Nevertheless, relapse of T2DM after an initial remission following bariatric surgery has also been a challenge [12, 19].

Although relapse of T2DM following initial remission after bariatric surgery has attracted more attention, it has been previously been difficult to accurately estimate the incidence because most studies did not report this outcome among patients who experienced an initial remission. However, to date, more and more studies reported the relapse rate after an initial remission, which provides sufficient data for accurately estimating the incidence of relapse of T2DM after RYBG surgery. We thereby performed the current meta-analysis to accurately estimate the long-term relapse rate of T2DM following RYGB procedure through combining the long-term results in published studies.

## Methods

We designed the present meta-analysis and subsequently reported all pooled findings according to the Preferred Reporting Items for Systematic Reviews and Meta-Analyses (PRISMA) statement [20]. Meanwhile, the Cochrane methodological framework was utilized to instruct performing our meta-analysis [21]. However, we did not register the formal protocol of our meta-analysis in any public platforms. No ethics approval and informed consent were required because all statistical analyses were performed based on published studies.

### Search Strategy

We electronically searched PubMed, Embase, and the Cochrane Library for the purpose of obtaining all potentially eligible studies from their inception to November 30, 2020, and the latest search was updated in July 2021. We used the following core keywords to construct the search strategy: T2DM, bariatric surgery, metabolic surgery, Roux-en-Y gastric bypass, RYGB, relapse, or recurrence. For individual database, we modified the search strategy according to the unique requirements in order to increase the sensitivity of the search strategy. Additionally, two investigators (Zhiqing Yu and Youcheng Zhang) manually reviewed the bibliographies of all included studies. When disagreement about identification of studies was detected, we invited a third senior investigator (Peiwu Li) to resolve it. Details of search strategies for target databases were summarized in Table S1.

### Selection Criteria

According to our aims, we developed the following selection criteria by using PICO acronym: (a) population (P), all adult patients were definitively diagnosed with T2DM according to the recognized standards; (b) interventions (I), all patients were treated with RYGB surgery; (c) outcome, all studies must report at least one of the following outcomes including the long-term relapse rate of T2DM, which was defined to be more than 5-year follow-up, remission rate, and HR for comparison of RYGB and SG; and (d) study design, randomized controlled trial (RCT), prospective cohort, and retrospective cohort were all considered to be eligible for our criteria. In the current meta-analysis, we only included full-text studies published in English.

Individual study was excluded if one of the following exclusion criteria was covered: (a) conference abstract; (b) duplicate report with insufficient information and poor methodological quality; and (c) ineligible design including case report, case series, experimental trials, and review.

### Data Extraction

In line with our aims, two independent investigators (Zhiqing Yu and Donghong Ma) extracted the following essential data by using the standard data extraction form: the name of all authors, year of publication, country where the study is performed, design of the study, sample size, proportion of male patients, number of patients who experienced long-term relapse of T2DM, number of patients who experienced remission and HR for comparison of RYGB and SG, and essential information for assessment of risk of bias. We invited a third senior investigator (Peirong Li) to resolve any disagreement about data extraction.

### Outcomes of Interest

In the current meta-analysis, we defined long-term relapse rate after initial remission as the primary outcome and initial remission rate which was defined as a fasting glucose concentration of 5.6 mmol/L or less and an HbA1c concentration of 65% or less (≤ 47.5 mmol/mol) without active pharmacological treatment for at least 1 year [22], with hazard ratio for comparison of RYGB, and SG as the secondary outcomes.

### Data Synthesis

To summarize the initial remission rate and long-term relapse rate, we firstly extracted the number of patients who underwent RYGB and then calculated the number of patients who experienced initial remission, which was used as the total sample size for the calculation of long-term relapse rate subsequently. Since there were 2 studies comparing the recurrence rate between RYGB and SG, we also extracted the hazard ratio for this comparison of RYGB and SG as one of the secondary analysis.

### Quality of the Evidence

In our meta-analysis, 1 RCT, 6 prospective cohort studies, and 10 retrospective cohort studies were included for analysis finally. Consequently, we used Cochrane risk of bias assessment tool to appraise the risk of bias of RCT and used the methodological items for non-randomized studies (MINORS) to assess the quality of the prospective or retrospective study. Quality assessment was performed by two independent authors (Peirong Li and Haidan Zhang). Discrepancy during quality assessment was resolved through consulting a third senior investigator (Zhiqing Yu).

### Statistical Analysis

Finally, we used STATA SE 14.0 software (StataCorp, College Station, Texas, USA) to perform all statistical analyses. We used odds ratio (OR) with corresponding 95% confidence intervals (CIs) to express all pooled results. Statistical heterogeneity across all eligible studies was evaluated by simultaneously using Cochran’s *Q* test and the *I*^2^ index, and an *I*^2^ of more than 50% and a *P* of less than 0.1 indicated statistical heterogeneity [23]. The random effects model was used to conduct statistical analysis because the variations among studies cannot be ignored [24]. We also designed subgroup analysis according to the study design and threshold of glycosylated hemoglobin A1c (HbA1c) for defining relapse. Moreover, we also examined the robustness of pooled estimates through performing sequential omission of each individual study [25]. A *P* value of < 0.05 was considered statistically different.

## Results

### Identification of Studies

**Fig. 1:**
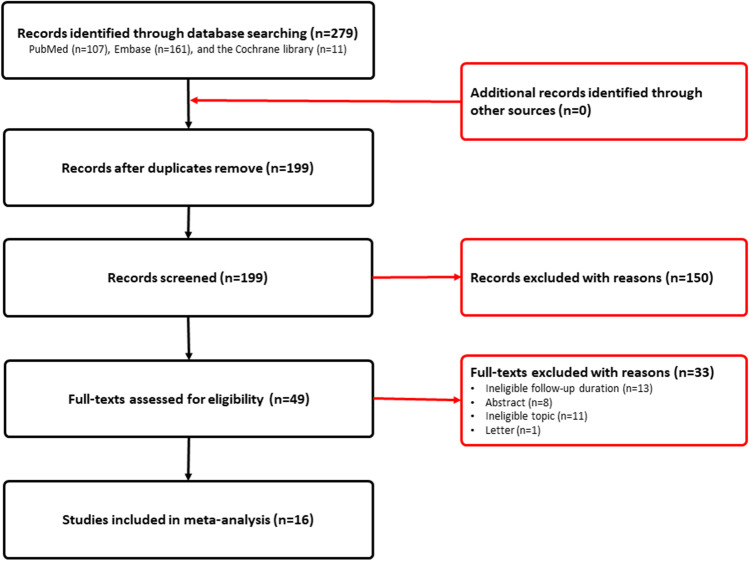
Flow diagram of identification and selection of eligible studies

We identified 279 studies after initially searching PubMed, Embase, and the Cochrane Library until to July 2021. A total of 199 studies remained after removing 80 duplicate studies. Then, a total of 150 ineligible studies were excluded after carefully screening titles and abstracts. Finally, we included 17 eligible studies [3, 19, 26–40] into the final statistical analysis after excluding 33 ineligible studies according to the following reasons: ineligible topic (*n* = 11), ineligible follow-up duration (*n* = 13), abstract (*n* = 8), and letter (*n* = 1). The process of identification and selection of eligible studies was displayed in Fig. 1.

### Characteristics of the Included Studies

Among the 17 included studies [3, 19, 26–40], most were performed in European countries. The sample size of receiving RYGB in individual study was between 19 and 4434 except for one study which did not report the number of patients underwent this procedure. The number of patients underwent initial remission in individual study was ranging from 9 to 2254. All studies reported the HbA1c threshold for confirming relapse of T2DM, and 10 studies introduced the definition of T2DM relapse. Details of characteristics of all included studies were summarized in Table 1.

**Table 1:** Basic characteristics of all included studies

Study, year	Country	Design	Number of patients received RYGB	Number of patients experienced remission	Definition of remission	Definition of relapse	HbA1c threshold for relapse, %	Male (%)
Mingrone, 2015	Italy	rct	19	15	6.5% HbA1c without taking active pharmacological treatment at least 1 year	HbA1c ≤ 6·5%	6.5	n.r
Chikunguwo, 2009	USA	pc	177	157	No requirement of taking diabetic medication or dietary at postoperative course	n.r	7.0	17.0
Debedat, 2018	France	pc	175	107	HbA1c < 6.5% without taking glucose-lowering agents at least 1 year	n.r	6.5	22.3
Dogan, 2014	Netherlands	pc	52	25 (27)	FPG < 5.6 mmol/L and HbA1c < 6.0% without taking glucose-lowering medication for at least 1 year	n.r	6.5	44.0
Elshaer, 2020	UK	pc	99	68	Recovery of glycemic status without hypoglycemic agents	n.r	6.5	30.0
Ghio, 2016	Spain	pc	24	9	HbA1c < 6.5% and FPG < 126 mg/dL without taking hypoglycemic agents	n.r	6.5	n.r
Nora, 2017	Portugal	pc	114	78	HbA1c < 6.5% without taking anti-diabetic drugs at least 6 months	n.r	6.5	11.4
Arterburn, 2012	USA	rsc	4434	2254	FPG < 100 and/or HbA1c < 6.0% lasting 90 days without taking anti-diabetic agents	HbA1c ≥ 6.5%, FPG ≥ 126 mg/dL, or taking anti-diabetic agents again	6.5	22.9
DiGiorgi, 2009	USA	rsc	124	100	n.r	HbA1c > 6.0%, FPG > 124 mg/dL or taking anti-diabetic agents	6.0	38.0
Hollande, 2020	Spain	rsc	562	410	HbA1c < 6.5% and FPG < 126 mg/dl without taking diabetic medication	Diabetes was confirmed again	6.5	30.8
Madsen, 2019	Denmark	rsc	786/1061	492	HbA1c < 6.5% without taking anti-diabetic agents or HbA1c < 6.0% without taking metformin	HbA1c ≥ 6.5% or GLD was prescribed again	6.5	36.5
McTigue, 2020	USA	rsc	n.r	2332	HbA1c < 6.5% at least 6 months	HbA1c ≥ 6.5% or taking anti-diabetic agents again	6.5	n.r
Oliverira, 2017	Brazil	rsc	254	208	HbA1c < 6.5% and FPG < 100–125 mg/dL at least 1 year without taking drugs	n.r	6.5	24.4
Wang, 2019	China	rsc	n.r	24	FBG < 5.6 mmol/L and HbA1c < 6.0% for at least 1 year without taking drugs	HbA1c ≥ 7.0%	6.0	70.8
Aminian, 2020	Spain	rsc	580	360	HbA1c < 6.5%, FBG < 126 mg/dL, without taking diabetes drugs	FBG ≥ 126 mg/dL or HbA1c ≥ 6.5% or without taking anti-diabetic drugs	6.5	n.r
Brethauer, 2013	USA	rsc	162	115	HbA1c < 6.5%, FBG < 125 mg/dL, without taking diabetes drugs	FBG ≥ 126 mg/dL or HbA1c ≥ 6.5% or without taking diabetes drugs	6.5	26.0
Conte, 2020a	France	rsc	101	n.r	Interruption of diabetes drugs for at least 6 months	Resumption of diabetes drugs after remission	n.r	n.r
Conte, 2020b	France	rsc	155	n.r	Interruption of diabetes drugs for at least 6 months	Resumption of diabetes drugs after remission	n.r	n.r

*RYGB*, Roux-en-Y gastric bypass; *T2DM*, type 2 diabetes mellitus; *HbA1C*, hemoglobin A1c; *FPG*, fasting plasma glucose; *rct*, randomized controlled trial; *pc*, prospective cohort; *rsc*, retrospective cohort; *n.r.*, not reported

### Methodological Quality

We included 1 RCT [36], 6 prospective cohorts [26, 28, 30–32, 37], and 10 retrospective cohorts [19, 27, 29, 33–36, 38–40] in the final analysis. Finally, RCT was rated as low quality based on Cochrane risk of bias, and the remaining 14 studies were identified as moderate-to-high quality because the total quality score of individual study based on the methodological items for non-randomized studies was between 7 and 9. We summarized the results of quality assessment in Table S2.

### Meta-analysis of Long-Term Relapse Rate After Initial Remission

**Fig. 2:**
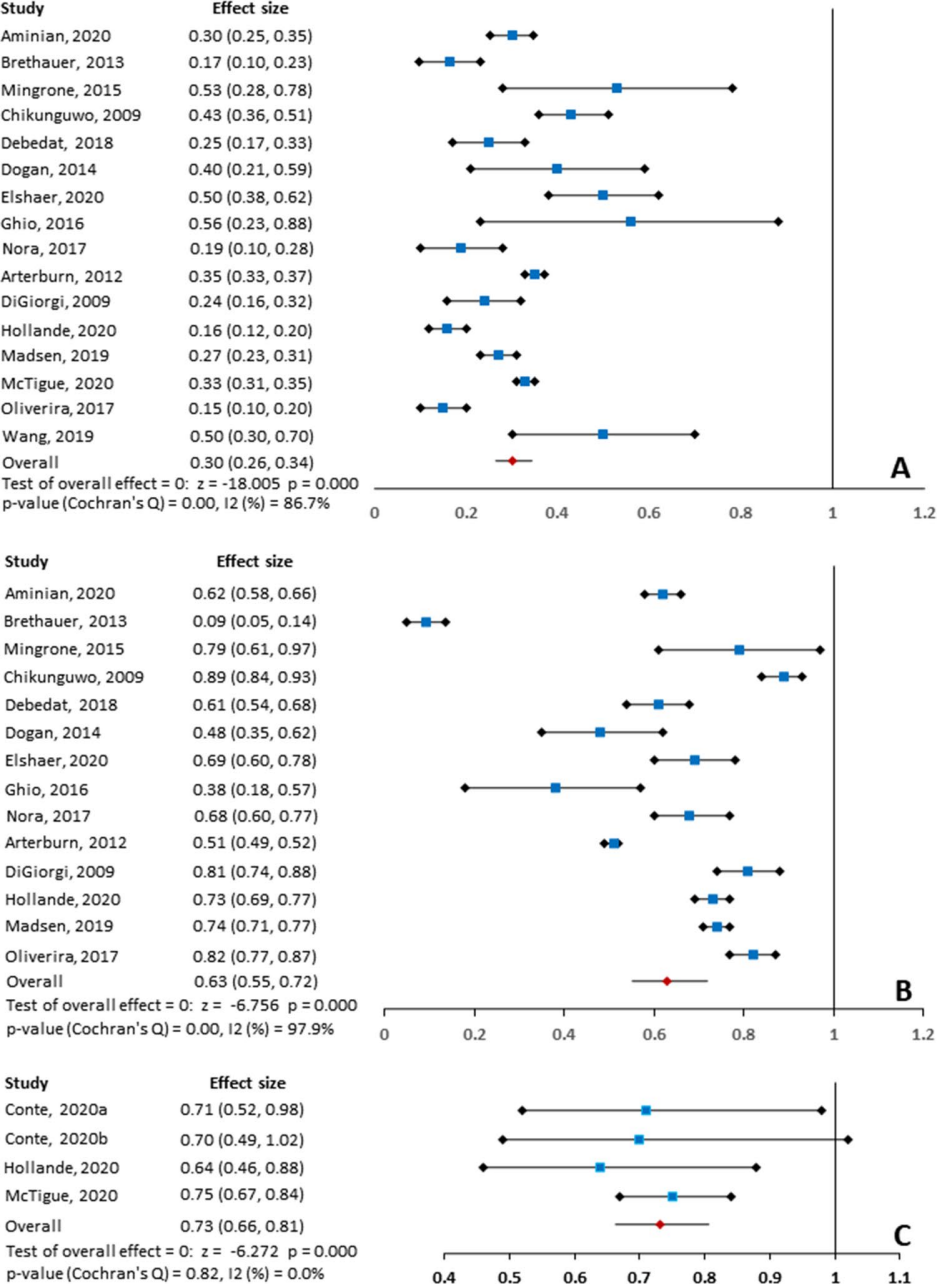
Meta-analysis of long-term relapse rate (**A**),initial remission rate (**B**), and the risk of recurrence for a comparison of RYGB and SG (**C**)

Among the included 17 studies, 16 studies reported the long-term relapse rate of T2DM after RYGB; the long-term relapse rate eventually reported in individual study was varying from 0.15 to 0.56 during the follow-up. Meta-analysis generated a long-term relapse rate of 0.30 (95% CI, 0.26, 0.34; *P* < 0.001, *I*^*2*^ = 86.7% [*P*_heterogeneity_ < 0.001]) after RYGB during follow-up. The result of individual study and pooled result was displayed in Fig. 2A.

Subgroup analysis according to HbA1c thresholds for defining long-term relapse of T2DM suggested that the long-term relapse rate was comparable between thresholds of 6.5 and 6.0%, with a pooled rate of 0.29 (95% CI, 0.25, 0.33; *P* < 0.001, *I*^*2*^ = 87.6% [*P*_heterogeneity_ < 0.001]) versus 0.34 (95% CI, 0.17, 0.70; *P* = 0.004, *I *^*2*^ = 85.5% [*P*_heterogeneity_ = 0.009]), respectively. However, the pooled rate was 0.43 (95% CI, 0.36, 0.51; *P* < 0.001) in the threshold of 7.0% group, which was higher than that in the threshold of 6.5% and 6.0% groups. Subgroup analysis based on HbA1c threshold was summarized in Table 2.

**Table 2:** Subgroup analysis of long-term relapse rate and initial remission rate of T2DM for patients underwent RYGB surgery

	*N*	Rate (95%CI)	*P*	*I* square	*P* (heterogeneity)
**Remission**	**13**	**0.63 (0.55, 0.72)**	** < 0.001**	**97.9**	** < 0.001**
*Study type*					
RCT	1	0.79 (0.63, 0.99)	0.046	n.a	n.a
Prospective cohort study	7	0.64 (0.53, 0.78)	< 0.001	92.6	< 0.001
Retrospective cohort study	5	0.61 (0.50, 0.73)	< 0.001	98.6	< 0.001
**Relapse**	**15**	**0.30 (0.26, 0.34)**	** < 0.001**	**86.7**	** < 0.001**
*Study type*					
RCT	1	0.53 (0.32,0.88)	0.015	n.a	n.a
Prospective cohort study	7	0.37 (0.27,0.49)	< 0.001	75.7	0.001
Retrospective cohort study	7	0.26 (0.23,0.31)	< 0.001	89.8	< 0.001
*Threshold of HbA1c*					
7.0%	3	0.4 3(0.36, 0.51)	< 0.001	n.a	n.a
6.5%	8	0.29 (0.25, 0.33)	< 0.001	94.4	< 0.001
6.0%	4	0.34(0.17, 0.70)	0.004	85.5	0.009

Subgroup analysis of long-term relapse according to study design suggested that the pooled rate based on prospective and retrospective studies was 0.37 (95% CI, 0.27, 0.49; *P* < 0.001, *I*^*2*^ = 75.7% [*P*_heterogeneity_ = 0.001]) and 0.26 (95% CI, 0.23, 0.31; *P* < 0.001, *I*^*2*^ = 89.8% [*P*_heterogeneity_ < 0.001]), which were all lower than that based on RCT, with a pooled rate of 0.53 (95% CI, 0.32, 0.88; *P* = 0.015). Subgroup analysis based on study design was summarized in Table 2.

### Meta-analysis of Initial Remission Rate After RYGB Surgery

Among the 17 included studies, 15 studies reported the initial remission rate after receiving RYGB surgery. The initial remission rate of individual study was ranging from 0.38 to 0.89, and meta-analysis revealed a pooled remission rate of 0.63 (95%CI, 0.55, 0.72; *P* < 0.001, *I*^*2*^ = 97.9% [*P*_heterogeneity_ < 0.001]). The pooled remission rate and remission rate of individual study were all displayed in Fig. 2B.

Subgroup analysis according to study design suggested that the initial remission rate reported by RCT was 0.79 (95% CI, 0.63, 0.99; *P* = 0.046), and the initial remission rate based on prospective and retrospective studies was 0.64 (95% CI, 0.53, 0.78; *P* < 0.001, *I*^*2*^ = 92.6% [*P*_heterogeneity_ < 0.01]) and 0.61 (95% CI, 0.50, 0.73; *P* < 0.001, *I*^*2*^ = 98.6% [*P*_heterogeneity_ < 0.001]), respectively. Subgroup analysis based on study design was summarized in Table 2.

### Meta-analysis of the Risk of Recurrence for Comparison of RYGB and SG

Among 17 eligible studies, 3 publications including 4 studies reported the hazard ratio of recurrence when RYGB surgery compared to SG surgery. Meta-analysis suggested a pooled hazard ratio of 0.73 (95% CI, 0.66, 0.81; *P* < 0.001, *I*^*2*^ = 0.0% [*P*_heterogeneity_ = 0.82]) for the comparison of RYGB and SG, indicating that RYGB was associated with a lower risk of recurrence of T2DM compared to SG. The pooled result was displayed in Fig. 2C.

### Sensitivity Analysis

**Fig. 3:**
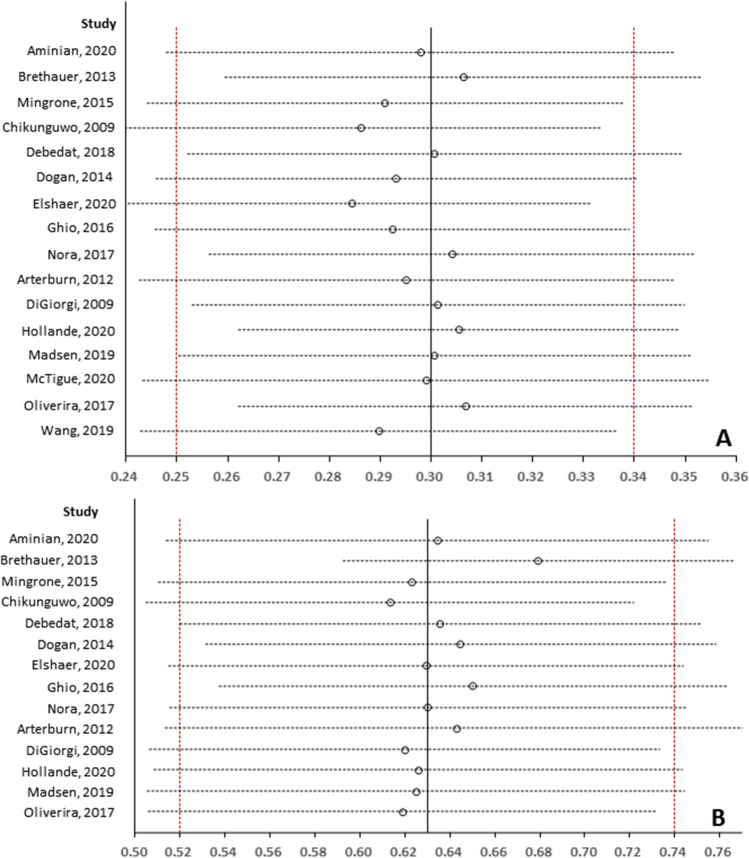
Sensitivity analysis of long-term relapse rate (**A**) and initial remission rate (**B**)

In order to examine the robustness of pooled results in terms of long-term relapse rate and initial remission rate, we conducted sensitivity analysis with the sequential omission of each individual study method, and sensitivity analysis suggested a robust pooled long-term relapse rate (see Fig. 3A) and initial remission rate (see Fig. 3B).

## Discussion

T2DM has been one of the major global health problems around the world due to the increase in the incidence of DM and obesity [2]. Patients with T2DM will experience several life-threatening conditions such as vascular diseases, which are the key contributor to higher medical expenditures, poor QoL, and higher mortality [1]. Considering the fact that conventional treatment regimens such as medical and excise therapies are not satisfactory for treating obese patients accompanied by DM [4–6], bariatric surgery is therefore developed and then widely used in clinical practice [5]. As one of the most common bariatric surgeries, RYGB has been frequently used to treat T2DM and achieved a promising initial remission of 60–75% [28]. However, more and more attention toward relapses of T2DM patients following initial remission after RYGB has been paid [18]. In the current meta-analysis, we included 17 eligible studies and then generated a pooled initial remission rate of 63.0% and a pooled long-term relapse rate of 30.0% in patients with T2DM who underwent RYGB surgery. Meanwhile, we also concluded that the risk of recurrence of T2DM in patients who received RYGB surgery was lower than that in patients who received SG surgery, with a pooled HR of 0.73.

To date, no meta-analysis focused on this topic has been published. The current meta-analysis firstly accumulated the long-term relapse rate following the initial remission after RYGB surgery. Our result suggested that 63.0% patients with T2DM achieved therapeutic goal after receiving RYGB surgery, which was similarly consistent with previous findings (60–75%) [28]. Moreover, subgroup analysis further established this result, with a pooled initial remission rate of 64.0% in prospective studies and 61.0% in retrospective studies. However, result from RCT (79.0%) was higher than previous results. It is important to emphasize that, in the current meta-analysis, only one RCT with extremely small sample size (19) was included [36], and thus, the result should be cautiously interpreted. Certainly, it is essential to perform more studies with RCT design to further answer this issue. Additionally, our meta-analysis suggested that 30.0% of patients who achieved initial remission experienced long-term relapse, which was consistent with most results reported previously, with a median rate of 30.0% [9, 19, 28]. Furthermore, our subgroup analyses based on prospective (37.0%) and retrospective (26.0%) studies also obtained consistent results with previous findings. However, the result of RCT obtained a relatively higher pooled rate of 53.0%. As explained above, this RCT might be underpowered by its insufficient sample size, and therefore, the long-term relapse rate from this study should be further examined. Moreover, subgroup analysis according to HbA1c threshold for defining long-term relapse was also conducted, and results based on 6.0% (34.0%) and 6.5% (29.0%) thresholds suggested consistent results with previous findings. However, the result based on the 7.0% threshold found a relatively higher long-term relapse (43.0%) compared to previous findings. As a result, it should be further investigated which level of HbA1c thresholds can be rationally used to define long-term relapse of T2DM after RYGB surgery.

Evidence obviously suggested that laparoscopic SG and gastric bypass (especially RYGB) have been regarded as the two most common bariatric surgeries used recently [41], and there are emerging evidence indicated that gastric bypass may achieve more higher remission and lower relapse rate compared to SG procedure because gastric bypass procedure may result in more durable weight loss and glycemic control [42]. In our meta-analysis, we also investigated the comparative hazard ratio when using RYGB compared to SG, and a pooled HR of 0.73 was generated, which further established the conclusion that RYGB surgery was associated with lower relapse compared to SG for the treatment of obese patients with T2DM.

Although our meta-analysis incorporated 17 eligible studies to obtain more reliable and robust results, some limitation should be further interpreted. Firstly, recent statements from the Diabetic Surgery Summit have indicated that bariatric surgery should be performed in T2DM patients with a body mass index (BMI) > 35 kg/m^2^ and may be an option for T2DM patients with a BMI 30–35 kg/m^2^ and major comorbidities [43], which means that patients with an unqualified BMI should not be included in our study. Secondly, we included studies with retrospective cohort design in our analysis because the sample size and the accumulated number of eligible studies were relatively small in those studies with the prospective cohort design, which will definitely undermine the quality of evidence of our analysis. To possibly avoid such effect, however, we designed subgroup analysis according to study design to further test robustness of pooled results. Thirdly, we excluded studies that only reported short-term relapse rate, which might introduce bias in the overall results.

## Conclusions

Based on the limited available evidence, we concluded that RYGB may be the preferred treatment option for the treatment of obese patients with T2DM because it was associated with satisfactory initial remission and relatively lower relapse rate. Meanwhile, RYGB may be superior to SG because of its associated relatively lower risk of recurrence of T2DM. Certainly, more studies with RCT design should be designed in order to further determine the initial remission and long-term relapse rate after RYGB surgery because only one RCT with extremely insufficient sample size was identified to date.

## Supplementary Information

Below is the link to the electronic supplementary material.

Supplementary file1 (DOCX 16 KB)

Supplementary file2 (DOCX 24 KB)
