# Supplementary material for: Meta-analysis of Long-Term Relapse Rate of Type 2 Diabetes Following Initial Remission After Roux-en-Y Gastric Bypass
Source: Obes Surg. 2021 Sep 10;31(11):5034–43. doi: 10.1007/s11695-021-05692-4 (PMC8490229; doi:10.1007/s11695-021-05692-4)
Supplement: Supplementary file 1 — Supplementary file1 (DOCX 16 KB) [file 11695_2021_5692_MOESM1_ESM.docx]

**Search query of PubMed**

| NO. | Query | Results |
| --- | --- | --- |
| #10 | #3 and #6 and #9 | 107 |
| #9 | #7 or #8 | 350,849 |
| #8 | ((((Recurrences[Title/Abstract]) OR (Recrudescence[Title/Abstract])) OR (Recrudescences[Title/Abstract])) OR (Relapse[Title/Abstract])) OR (Relapses[Title/Abstract]) | 198,927 |
| #7 | "Recurrence"[Mesh] | 190,434 |
| #6 | #4 or #5 | 35,035 |
| #5 | ((((((((((((Greenville Gastric Bypass[Title/Abstract]) OR (Gastrojejunostomy[Title/Abstract])) OR (Gastrojejunostomies[Title/Abstract])) OR (Roux-en-Y Gastric Bypass[Title/Abstract])) OR (Roux en Y Gastric Bypass[Title/Abstract])) OR (Gastroileal Bypass[Title/Abstract])) OR (Metabolic Surgery[Title/Abstract])) OR (Metabolic Surgeries[Title/Abstract])) OR (Bariatric Surgical Procedures[Title/Abstract])) OR (Bariatric Surgical Procedure[Title/Abstract])) OR (Bariatric Surgeries[Title/Abstract])) OR (Stomach Stapling[Title/Abstract])) OR (Sleeve gastrectomy[Title/Abstract]) | 16,304 |
| #4 | ("Gastric Bypass"[Mesh]) OR "Bariatric Surgery"[Mesh] | 28,818 |
| #3 | #1 or #2 | 200,337 |
| #2 | ((((((((((((((Ketosis-Resistant Diabetes Mellitus[Title/Abstract]) OR (Non-Insulin-Dependent Diabetes Mellitus[Title/Abstract])) OR (Stable Diabetes Mellitus[Title/Abstract])) OR (NIDDM[Title/Abstract])) OR (Maturity-Onset Diabetes Mellitus[Title/Abstract])) OR (Maturity Onset Diabetes Mellitus[Title/Abstract])) OR (MODY[Title/Abstract])) OR (Slow-Onset Diabetes Mellitus[Title/Abstract])) OR (Type 2 Diabetes Mellitus[Title/Abstract])) OR (Noninsulin-Dependent Diabetes Mellitus[Title/Abstract])) OR (Noninsulin Dependent Diabetes Mellitus[Title/Abstract])) OR (Maturity-Onset Diabetes[Title/Abstract])) OR (Maturity Onset Diabetes[Title/Abstract])) OR (Type 2 Diabetes[Title/Abstract])) OR (Adult-Onset Diabetes Mellitus[Title/Abstract]) | 151,937 |
| #1 | "Diabetes Mellitus, Type 2"[Mesh] | 144,056 |

**Search query of Embase (via OVID)**

| NO. | Query | Results |
| --- | --- | --- |
| #1 | (Ketosis-Resistant Diabetes Mellitus or Non-Insulin-Dependent Diabetes Mellitus or Stable Diabetes Mellitus or NIDDM or Maturity-Onset Diabetes Mellitus or Maturity Onset Diabetes Mellitus or MODY or Slow-Onset Diabetes Mellitus or Type 2 Diabetes Mellitus or Noninsulin-Dependent Diabetes Mellitus or Noninsulin Dependent Diabetes Mellitus or Maturity-Onset Diabetes or Maturity Onset Diabetes or Type 2 Diabetes or Adult-Onset Diabetes Mellitus).af. | 328128 |
| #2 | (Diabetes Mellitus, Type 2 or Type 2 Diabetes Mellitus).sh. | 3173 |
| #3 | #1 or #2 | 329231 |
| #4 | (Greenville Gastric Bypass or Gastrojejunostomy or Gastrojejunostomies or Roux-en-Y Gastric Bypass or Roux en Y Gastric Bypass or Gastroileal Bypass or Metabolic Surgery or Metabolic Surgeries or Bariatric Surgical Procedures or Bariatric Surgical Procedure or Bariatric Surgeries or Stomach Stapling or Sleeve gastrectomy).af. | 37073 |
| #5 | (Gastric Bypas or Bariatric Surgery).sh. | 34923 |
| #6 | #4 or #5 | 59168 |
| #7 | (Recurrences or Recrudescenc or Recrudescences or Relapse or Relapses).af. | 377593 |
| #8 | (Recurrence or Relapse).sh. | 157132 |
| #9 | #7 or #8 | 377604 |
| #10 | #3 and #6 and #9 | 161 |

**Search query of Cochrane library**

| No | Query | Results |
| --- | --- | --- |
| #1 | (Ketosis-Resistant Diabetes Mellitus):ti,ab,kw OR (Non-Insulin-Dependent Diabetes Mellitus):ti,ab,kw OR (Stable Diabetes  Mellitus):ti,ab,kw OR (NIDDM):ti,ab,kw OR (Maturity-Onset Diabetes Mellitus):ti,ab,kw | 21021 |
| #2 | (Maturity Onset Diabetes Mellitus):ti,ab,kw OR (MODY):ti,ab,kw OR (Slow-Onset Diabetes Mellitus):ti,ab,kw OR (Type 2 Diabetes  Mellitus):ti,ab,kw OR (Noninsulin-Dependent Diabetes Mellitus):ti,ab,kw | 43217 |
| #3 | (Noninsulin Dependent Diabetes Mellitu):ti,ab,kw OR (Maturity-Onset Diabetes):ti,ab,kw OR (Maturity Onset Diabetes):ti,ab,kw  OR (Type 2 Diabetes):ti,ab,kw OR (Adult-Onset Diabetes Mellitus):ti,ab,kw | 49094 |
| #4 | MeSH descriptor: [Diabetes Mellitus, Type 2] explode all trees | 18434 |
| #5 | #1 or #2 or #3 or #4 | 51926 |
| #6 | (Greenville Gastric Bypass):ti,ab,kw OR (Gastrojejunostomy):ti,ab,kw OR (Gastrojejunostomies):ti,ab,kw OR (Roux-en-Y Gastric  Bypass):ti,ab,kw OR (Roux en Y Gastric Bypass):ti,ab,kw | 1385 |
| #7 | (Gastroileal Bypass):ti,ab,kw OR (Metabolic Surgery):ti,ab,kw OR (Metabolic Surgeries):ti,ab,kw OR (Bariatric Surgical  Procedures):ti,ab,kw OR (Bariatric Surgical Procedure):ti,ab,kw | 3552 |
| #8 | (Bariatric Surgeries):ti,ab,kw OR (Stomach Stapling):ti,ab,kw OR (Sleeve gastrectomy):ti,ab,kw | 1240 |
| #9 | MeSH descriptor: [Gastric Bypass] explode all trees | 516 |
| #10 | MeSH descriptor: [Bariatric Surgery] explode all trees | 1067 |
| #11 | #6 or #7 or #8 or #9 or #10 | 5689 |
| #12 | (Recurrences):ti,ab,kw OR (Recrudescence):ti,ab,kw OR (Recrudescences):ti,ab,kw OR (Relapse):ti,ab,kw OR (Relapses):ti,ab,kw | 36949 |
| #13 | MeSH descriptor: [Recurrence] explode all trees | 12390 |
| #14 | #12 or #13 | 45396 |
| #15 | #5 and #11 and #14 | 11 |
