# Supplementary material for: Meta-analysis of Long-Term Relapse Rate of Type 2 Diabetes Following Initial Remission After Roux-en-Y Gastric Bypass
Source: Obes Surg. 2021 Sep 10;31(11):5034–43. doi: 10.1007/s11695-021-05692-4 (PMC8490229; doi:10.1007/s11695-021-05692-4)
Supplement: Supplementary file 2 — Supplementary file2 (DOCX 24 KB) [file 11695_2021_5692_MOESM2_ESM.docx]

**Table S2.** The quality of all included studies.

| **Cochrane risk of bias assessment tool for randomized controlled trial** | | | | | | | | | | | | | | | | |
| --- | --- | --- | --- | --- | --- | --- | --- | --- | --- | --- | --- | --- | --- | --- | --- | --- |
| Study | | 1 | | | 2 | | 3 | | | 4 | | 5 | | 6 | | 7 |
| Mingrone, 2015 | | Low | | | Low | | Low | | | Low | | Low | | Low | | Low |
| 1. Random sequence generation (selection bias); 2. Allocation concealment (selection bias); 3. Blinding of participants and peraonnel (performance bias); 4. Blinding of outcome assessmnet (detection bias); 5. Incomplete outcome data (attrition bias); 6. Selective reporting (reporting bias); 7. Other bias. | | | | | | | | | | | | | | | | |
| **NOS criteria for cohort study** | | | | | | | | | | | | | | | | |
| Study | 1 | | 2 | 3 | | 4 | | 5 | 6 | | 7 | | 8 | | Total quality scores | |
| Chikunguwo, 2009 | ★ | | \ | ★ | | ★ | | ★ | ★ | | ★ | | ★ | | 7 | |
| Debedat, 2018 | ★ | | ★ | ★ | | ★ | | ★★ | ★ | | ★ | | ★ | | 9 | |
| Dogan, 2014 | ★ | | \ | ★ | | ★ | | ★ | ★ | | ★ | | ★ | | 7 | |
| Elshaer, 2020 | ★ | | ★ | ★ | | ★ | | ★★ | ★ | | ★ | | ★ | | 9 | |
| Ghio, 2016 | ★ | | ★ | ★ | | ★ | | ★★ | ★ | | ★ | | ★ | | 9 | |
| Nora, 2017 | ★ | | ★ | ★ | | ★ | | ★★ | ★ | | ★ | | ★ | | 9 | |
| Arterburn, 2012 | ★ | | \ | ★ | | ★ | | ★ | ★ | | ★ | | ★ | | 7 | |
| DiGiorgi, 2009 | ★ | | ★ | ★ | | ★ | | ★★ | ★ | | ★ | | ★ | | 9 | |
| Hollande, 2020 | ★ | | ★ | ★ | | ★ | | ★★ | ★ | | ★ | | ★ | | 9 | |
| Madsen, 2019 | ★ | | ★ | ★ | | ★ | | ★★ | ★ | | ★ | | \ | | 8 | |
| McTigue, 2020 | ★ | | ★ | ★ | | ★ | | ★★ | ★ | | ★ | | \ | | 8 | |
| Oliveira, 2017 | ★ | | \ | ★ | | ★ | | ★ | ★ | | ★ | | ★ | | 7 | |
| Wang, 2019 | ★ | | ★ | ★ | | ★ | | ★★ | ★ | | ★ | | ★ | | 9 | |
| Aminian, 2020 | ★ | | \ | ★ | | ★ | | ★ | ★ | | ★ | | ★ | | 7 | |
| Brethauer, 2013 | ★ | | \ | ★ | | ★ | | ★ | ★ | | ★ | | ★ | | 7 | |
| Conte, 2020 | ★ | | ★ | ★ | | ★ | | ★★ | ★ | | ★ | | ★ | | 9 | |
| 1. Representativeness of the exposed cohort; 2. Selection of the non-exposed cohort; 3. Ascertainment of exposure; 4. Demonstration that outcome of interest was not present at start of study; 5. Comparability of cohorts on the basis of the design or analysis; 6. Assessment of outcome; 7. Was follow-up long enough for outcomes to occur; 8. Adequacy of follow up of cohorts. | | | | | | | | | | | | | | | | |
